# 631 Impact of Cannabis Use on Outcomes in Burn Patients: A National Trauma Data Bank Analysis

**DOI:** 10.1093/jbcr/iraf019.260

**Published:** 2025-04-01

**Authors:** Eloise Stanton, Artur Manasyan, Haig Yenikomshian, Justin Gillenwater, Maxwell Johnson

**Affiliations:** University of Southern California; University of California Keck School of Medicine; University of California Keck School of Medicine; Los Angeles General Medical Center; University of California Keck School of Medicine

## Abstract

**Introduction:**

Cannabis use has increased with expanding legalization but is poorly studied in burn care. With known effects on pain perception, metabolism, and immune modulation, cannabis use may influence various aspects of burn treatment, including pain management, nutritional needs, and wound healing. This study aims to explore trends in cannabis use among burn patients and evaluate its association with clinical outcomes using the National Trauma Data Bank (NTDB).

**Methods:**

The NTDB was queried to identify burn patients from 2017 to 2022. Cannabis use was documented at admission via urine toxicology screens. Primary outcomes included mortality, stroke, myocardial infarction, organ failure, timing of surgery, and post-surgical complications. Secondary outcomes included emergency department vital signs, length of stay, and need for intensive care. Multivariable regression models were used for analysis while controlling for covariates including age, sex, %TBSA, and inhalation injury.

**Results:**

A total of 319,941 burn patients were identified in the NTDB during the study period, of which 52,803 (16.5%) had positive urine drug screens for cannabinoids upon admission. These patients were significantly more likely to be male (18% vs. 11%, p< 0.001) and were significantly younger (28.9 vs. 32.6 years old, p< 0.001). They were more likely to experience a venous thromboembolic event, required longer periods of intubation, were more likely to contract ventilator-associated pneumonia, required more surgical debridements/graftings, and overall had longer length of stay (p< 0.05). They were more likely to have comorbid substance use disorders and required significantly higher levels of sedatives. Multivariate regression demonstrated that patients with positive-cannabinoid screens had a significantly longer length of stay, intubation periods, and required a greater number of operative interventions (p< 0.05).

**Conclusions:**

This study found that burn patients with positive cannabis screens had longer hospital stays and higher rates of surgical interventions compared to those without cannabis use. Additionally, many cannabis-positive patients had comorbid substance use disorders, suggesting a complex interplay between cannabis use and overall health outcomes. The findings indicate the need for further investigation into how cannabis and other substance use may affect burn injuries and recovery, emphasizing the importance of comprehensive assessments in this patient population.

**Applicability of Research to Practice:**

This study highlights the need for burn care providers to consider cannabis use as a potential factor influencing and worsening patient outcomes. Integrating cannabis use screening into standard assessments and tailoring management strategies can help optimize care for burn patients. Further research on the role of cannabis in burn recovery is essential for developing evidence-based guidelines.

**Funding for the Study:**

N/A

